# Phase transfer entropy: a novel measure for effective connectivity among neuronal oscillations

**DOI:** 10.1186/1471-2202-14-S1-P305

**Published:** 2013-07-08

**Authors:** Felix Siebenhuehner, Muriel Lobier, Satu Palva, Matias Palva

**Affiliations:** 1 Neuroscience Center, University of Helsinki, Finland

## 

Phase synchronization of neuronal oscillations has been suggested to underlie the coordination and integration of anatomically distributed processing [1,2]. To quantify "causal" or directional inter-areal phase-phase interactions, a phase-based measure of effective connectivity is needed. Methods for detecting effective connectivity can be divided into model-based (*e.g*., Dynamic Causal Modeling [3]) and model-free techniques (*e.g*., Granger Causality [4]). Transfer Entropy (TE) [5] is a model-free measure of effective connectivity based on information theory. Prior implementations of TE, however, focus on real-valued time series where the signal amplitude is a major bias and phase is only an implicit variable. Furthermore, the robustness of existing TE methods to narrow-band filtering, noise, and linear mixing between signals is limited. Finally, current approaches are dependent on accurate *a priori *estimation of several parameters, which is not feasible for connectomics approaches with all-to-all mappings of inter-areal interactions. Here we advance a novel measure, Phase Transfer Entropy (Phase TE), to estimate directional connectivity between complex phase time series of filtered signals.

In this study, we first assess the reliability of Phase TE in quantifying directional connectivity. Second, we compare its performance to other TE implementations using real-valued narrowband and broadband signals. To quantify a unidirectional effect of signal 1 to signal 2, we define differential TE (dTE) as: dTE_(1→2) _= TE_(1→2) _- TE_(2→1) _and determine the sensitivity and specificity of our methods from the dTE distributions of uncoupled and coupled signals.

We simulated ecologically valid neuronal-like oscillations with coupled Neural Mass Models [6] and estimated the phase time series with Morlet filtering. Phase TE increased monotonically with coupling strength and discriminated between coupled and non-coupled time series. Phase TE was robust to realistic amounts of noise and/or linear mixing. Effective connectivity even with small coupling values was reliably detected for moderate signal-to-noise ratios using an amount of data commonly acquired in neuroimaging experiments. We found that Phase dTE did not yield false positives in the presence of mixing and/or noise. Across a range of noise and mixing values, the sensitivity of Phase TE was comparable to or better than the sensitivities of prior TE implementations. Finally, Phase TE was computationally much faster than prior TE implementations.

In conclusion, Phase TE is a computationally efficient method for detecting directed interactions between band-limited activities in neurophysiological time series. Phase TE works well with filtering and is robust against noise and linear mixing, which are the elementary confounders in electrophysiological data. Given that Phase TE is also essentially parameter free, we propose it to be an efficient and reliable method for assessing effective connectivity in connectomics analyses.
